# Satellite mega-constellations create risks in Low Earth Orbit, the atmosphere and on Earth

**DOI:** 10.1038/s41598-021-89909-7

**Published:** 2021-05-20

**Authors:** Aaron C. Boley, Michael Byers

**Affiliations:** 1 Department of Physics and Astronomy, The University of British Columbia, Vancouver, Canada; 2 Department of Political Science, The University of British Columbia, Vancouver, Canada

**Keywords:** Sustainability, Environmental impact, Aerospace engineering

## Abstract

The rapid development of mega-constellations risks multiple tragedies of the commons, including tragedies to ground-based astronomy, Earth orbit, and Earth’s upper atmosphere. Moreover, the connections between the Earth and space environments are inadequately taken into account by the adoption of a consumer electronic model applied to space assets. For example, we point out that satellite re-entries from the Starlink mega-constellation alone could deposit more aluminum into Earth’s upper atmosphere than what is done through meteoroids; they could thus become the dominant source of high-altitude alumina. Using simple models, we also show that untracked debris will lead to potentially dangerous on-orbit collisions on a regular basis due to the large number of satellites within mega-constellation orbital shells. The total cross-section of satellites in these constellations also greatly increases the risk of impacts due to meteoroids. De facto orbit occupation by single actors, inadequate regulatory frameworks, and the possibility of free-riding exacerbate these risks. International cooperation is urgently needed, along with a regulatory system that takes into account the effects of tens of thousands of satellites.

## Introduction

Companies are placing satellites into orbit at an unprecedented frequency to build ‘mega-constellations’ of communications satellites in Low Earth Orbit (LEO). In two years, the number of active and defunct satellites in LEO has increased by over 50%, to about 5000 (as of 30 March 2021). SpaceX alone is on track to add 11,000 more as it builds its Starlink mega-constellation and has already filed for permission for another 30,000 satellites with the Federal Communications Commission (FCC)^1^. Others have similar plans, including OneWeb, Amazon, Telesat, and GW, which is a Chinese state-owned company^2^. The current governance system for LEO, while slowly changing, is ill-equipped to handle large satellite systems. Here, we outline how applying the consumer electronic model to satellites could lead to multiple tragedies of the commons. Some of these are well known, such as impediments to astronomy and an increased risk of space debris, while others have received insufficient attention, including changes to the chemistry of Earth’s upper atmosphere and increased dangers on Earth’s surface from re-entered debris. The heavy use of certain orbital regions might also result in a de facto exclusion of other actors from them, violating the 1967 Outer Space Treaty. All of these challenges could be addressed in a coordinated manner through multilateral law-making, whether in the United Nations, the Inter-Agency Debris Committee (IADC), or an ad hoc process, rather than in an uncoordinated manner through different national laws. Regardless of the law-making forum, mega-constellations require a shift in perspectives and policies: from looking at single satellites, to evaluating systems of thousands of satellites, and doing so within an understanding of the limitations of Earth’s environment, including its orbits.

Thousands of satellites and 1500 rocket bodies provide considerable mass in LEO, which can break into debris upon collisions, explosions, or degradation in the harsh space environment. Fragmentations increase the cross-section of orbiting material, and with it, the collision probability per time. Eventually, collisions could dominate on-orbit evolution, a situation called the Kessler Syndrome^3^. There are already over 12,000 trackable debris pieces in LEO, with these being typically 10 cm in diameter or larger. Including sizes down to 1 cm, there are about a million inferred debris pieces, all of which threaten satellites, spacecraft and astronauts due to their orbits crisscrossing at high relative speeds. Simulations of the long-term evolution of debris suggest that LEO is already in the protracted initial stages of the Kessler Syndrome, but that this could be managed through active debris removal^4^. The addition of satellite mega-constellations and the general proliferation of low-cost satellites in LEO stresses the environment further^5–8^.

## Results

### The overall setting

**Figure 1:**
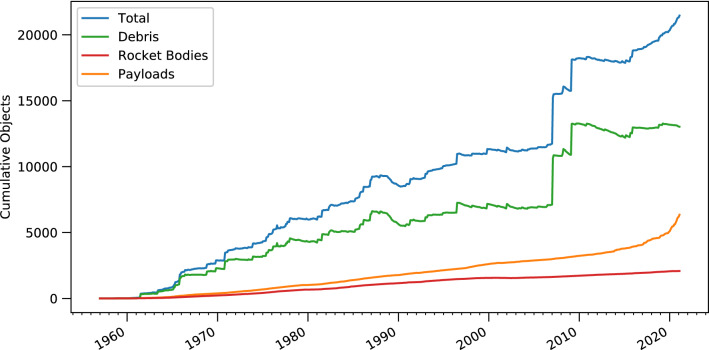
Cumulative on-orbit distribution functions (all orbits). Deorbited objects are not included. The 2007 and 2009 spikes are a Chinese anti-satellite test and the Iridium 33-Kosmos 2251 collision, respectively. The recent, rapid rise of the orange curve represents NewSpace (see "Methods").

The rapid development of the space environment through mega-constellations, predominately by the ongoing construction of Starlink, is shown by the cumulative payload distribution function (Fig. 1). From an environmental perspective, the slope change in the distribution function defines NewSpace, an era of dominance by commercial actors. Before 2015, changes in the total on-orbit objects came principally from fragmentations, with effects of the 2007 Chinese anti-satellite test and the 2009 Kosmos-2251/Iridium-33 collisions being evident on the graph.

**Figure 2:**
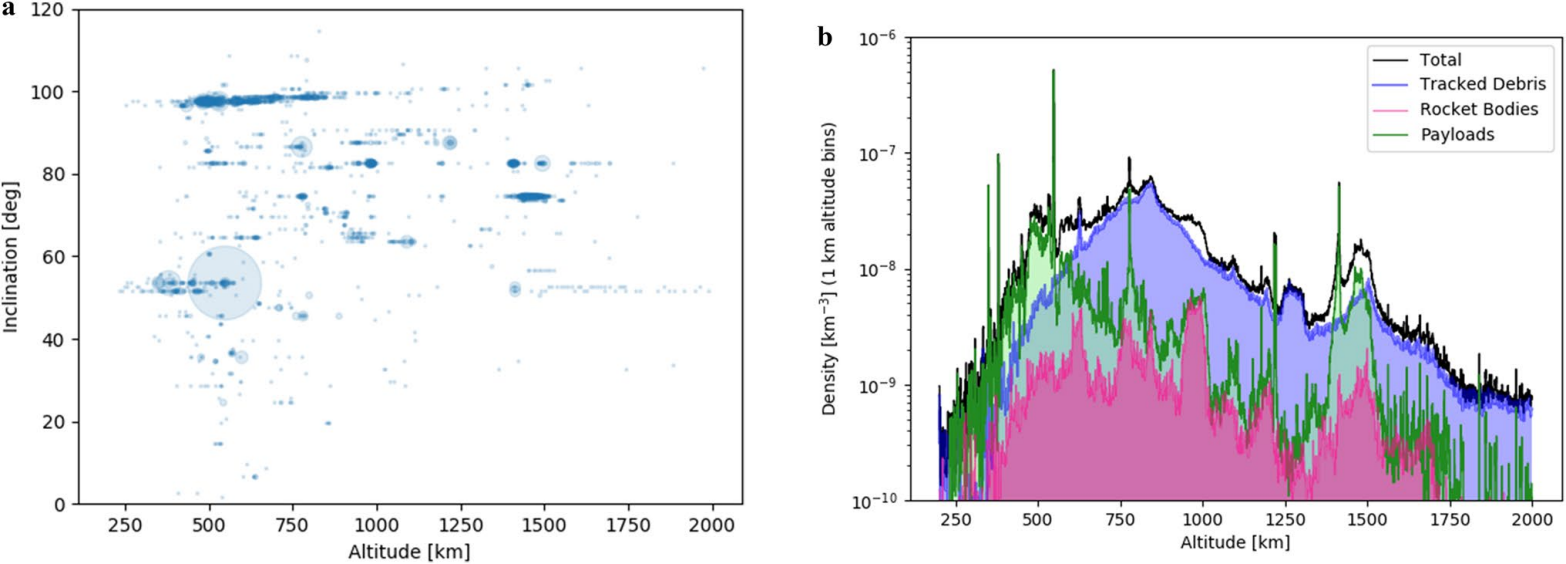
Orbital distribution and density information for objects in Low Earth Orbit (LEO). (Left) Distribution of payloads (active and defunct satellites), binned to the nearest 1 km in altitude and 1° in orbital inclination. The centre of each circle represents the position on the diagram, and the size of the circle is proportional to the number of satellites within the given parameter space. (Right) Number density of different space resident objects (SROs) based on 1 km radial bins, averaged over the entire sky. Because SRO objects are on elliptical orbits, the contribution of a given object to an orbital shell is weighted by the time that object spends in the shell. Despite significant parameter space, satellites are clustered in their orbits due to mission requirements. The emerging Starlink cluster at 550 km and 55° inclination is already evident in both plots (Left and Right).

Although the volume of space is large, individual satellites and satellite systems have specific functions, with associated altitudes and inclinations (Fig. 2). This increases congestion and requires active management for station keeping and collision avoidance^9^, with automatic collision-avoidance technology still under development. Improved space situational awareness is required, with data from operators as well as ground- and space-based sensors being widely and freely shared^10^. Improved communications between satellite operators are also necessary: in 2019, the European Space Agency moved an Earth observation satellite to avoid colliding with a Starlink satellite, after failing to reach SpaceX by e-mail. Internationally adopted ‘right of way’ rules are needed^10^ to prevent games of ‘chicken’, as companies seek to preserve thruster fuel and avoid service interruptions. SpaceX and NASA recently announced^11^ a cooperative agreement to help reduce the risk of collisions, but this is only one operator and one agency.

When completed, Starlink will include about as many satellites as there are trackable debris pieces today, while its total mass will equal all the mass currently in LEO—over 3000 tonnes. The satellites will be placed in narrow orbital shells, creating unprecedented congestion, with 1258 already in orbit (as of 30 March 2021). OneWeb has already placed an initial 146 satellites, and Amazon, Telesat, GW and other companies, operating under different national regulatory regimes, are soon likely to follow.

### Enhanced collision risk

**Figure 3:**
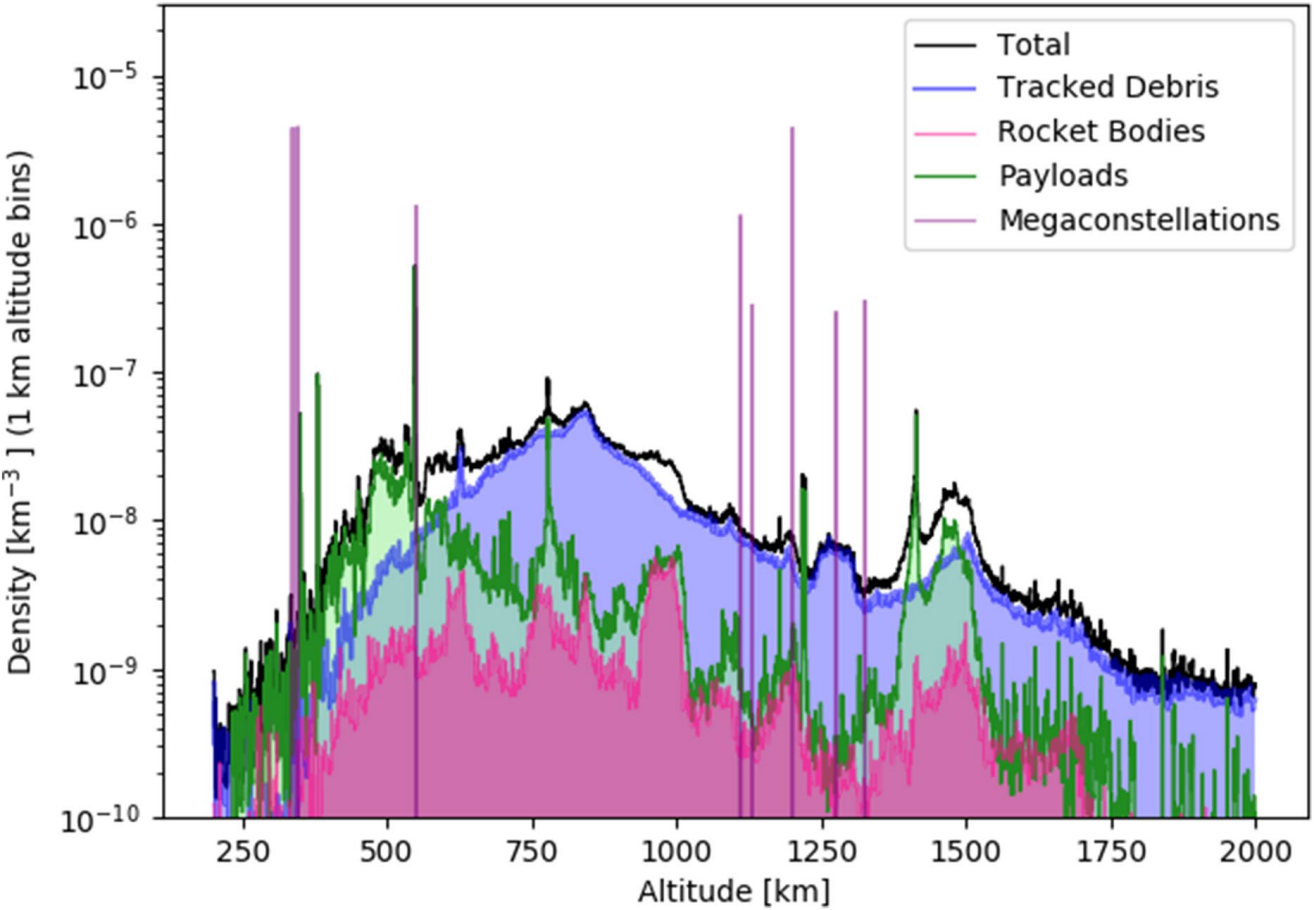
Satellite density distribution in LEO with the Starlink and OneWeb mega-constellations as filed (and amended) with the FCC. Provided that the orbits are nearly circular, the number densities in those shells will exceed 10^–6^ km^−3^. Because the collisional cross-section in those shells is also high, they represent regions that have a high collision risk whenever debris is too small to be tracked or collision avoidance manoeuvres are impossible for other reasons.

Mega-constellations are composed of mass-produced satellites with few backup systems. This consumer electronic model allows for short upgrade cycles and rapid expansions of capabilities, but also considerable discarded equipment. SpaceX will actively de-orbit its satellites at the end of their 5–6-year operational lives. However, this process takes 6 months, so roughly 10% will be de-orbiting at any time. If other companies do likewise, thousands of de-orbiting satellites will be slowly passing through the same congested space, posing collision risks. Failures will increase these numbers, although the long-term failure rate is difficult to project. Figure 3 is similar to the righthand portion of Fig. 2 but includes the Starlink and OneWeb mega-constellations as filed (and amended) with the FCC (see “Methods”). The large density spikes show that some shells will have satellite number densities in excess of $n={10}^{-6}{\text{ km}}^{-3}$.

Deorbiting satellites will be tracked and operational satellites can manoeuvre to avoid close conjunctions. However, this depends on ongoing communication and cooperation between operators, which at present is ad hoc and voluntary. A recent letter^12^ to the FCC from SpaceX suggests that some companies might be less-than-fully transparent about events^13^ in LEO.

Despite the congestion and traffic management challenges, FCC filings by SpaceX suggest that collision avoidance manoeuvres can in fact maintain collision-free operations in orbital shells and that the probability of a collision between a non-responsive satellite and tracked debris is negligible. However, the filings do not account for *untracked* debris^6^, including untracked debris decaying through the shells used by Starlink. Using simple estimates (see “Methods”), the probability that a single piece of untracked debris will hit any satellite in the Starlink 550 km shell is about 0.003 after one year. Thus, if at any time there are 230 pieces of untracked debris decaying through the 550 km orbital shell, there is a 50% chance that there will be one or more collisions between satellites in the shell and the debris. As discussed further in “Methods”, such a situation is plausible. Depending on the balance between the de-orbit and the collision rates, if subsequent fragmentation events lead to similar amounts of debris within that orbital shell, a runaway cascade of collisions could occur.

Fragmentation events are not confined to their local orbits, either. The India 2019 ASAT test was conducted at an altitude below 300 km in an effort to minimize long-lived debris. Nevertheless, debris was placed on orbits with apogees in excess of 1000 km. As of 30 March 2021, three tracked debris pieces remain in orbit^14^. Such long-lived debris has high eccentricities, and thus can cross multiple orbital shells twice per orbit. A major fragmentation event from a single satellite could affect all operators in LEO.

Even if debris collisions were avoidable, meteoroids are always a threat. The cumulative meteoroid flux^15^ for masses m > 10^–2^ g is about 1.2 × 10^–4^ meteoroids m^−2^ year^−1^ (see “Methods”). Such masses could cause non-negligible damage to satellites^16^. Assuming a Starlink constellation of 12,000 satellites (i.e. the initial phase), there is about a 50% chance of 15 or more meteoroid impacts per year at m > 10^–2^ g. Satellites will have shielding, but events that might be rare to a single satellite could become common across the constellation.

One partial response to these congestion and collision concerns is for operators to construct mega-constellations out of a smaller number of satellites. But this does not, individually or collectively, eliminate the need for an all-of-LEO approach to evaluating the effects of the construction and maintenance of any one constellation.

### Surface impacts and atmospheric effects

Although failures do occur, first stages of SpaceX rockets are usually landed and re-used, while second stages are usually controlled through re-entry and deposited in remote areas of ocean. This best practice might not be followed by others. For example, the first stages of the Soyuz rockets employed by OneWeb are not reusable, nor are the second stage re-entries controllable. The Long March rockets that will likely be employed by GW are similar. Uncontrolled re-entries do not always meet safety standards^17^, a situation that may be exacerbated by mega-constellations. Moreover, the cumulative impact of thousands of rocket stages on the ocean environment could be significant should those stages contain hazardous materials, such as unspent hydrazine fuels^17–19^. In the 1990s, Pacific island countries opposed the Sea Launch project because of environmental concerns, including from discarded rocket stages^20^. In 2016, Inuit in the Canadian Arctic protested the Russian practice of disposing rocket stages in the North Water Polynya, a biologically rich area of year-round open water^21^.

The first Starlink satellites contained some components that survive re-entry, with the highest human casualty risk for a single satellite calculated to be 1:17,400^22^, below NASA’s recommended 1:10,000 threshold. However, the initial approval process did not account for the cumulative casualty risk, and if all the then-planned 12,000 satellites had contained the same components, a continuous 5-year replacement cycle would have seen a 45% probability of one or more casualties per cycle. When the subsequent FCC petition process identified the problem, SpaceX reportedly replaced some materials with a view to having all of the satellite components now demise in the atmosphere^23^. Other companies, based in other countries, might not follow this best practice or be required to do so.

The demise of satellite components during re-entry introduces a different problem, since none of that material actually disappears. Starlink satellites have a dry mass of about 260 kg; 12,000 satellites will total 3100 tonnes. A 5-year cycle would see on average almost 2 tonnes re-entering Earth’s atmosphere daily. While small compared to the 54 daily tonnes of meteoroid mass^24^, the satellites are mostly aluminum; most meteoroids, in contrast, contain less than 1% Al by mass^25^. Thus, depending on the atmospheric residence time of material from re-entered satellites, each mega-constellation will produce fine particulates that could greatly exceed natural forms of high-altitude atmospheric aluminum deposition, particularly if the full numbers of envisaged satellites are launched. Anthropogenic deposition of aluminum in the atmosphere has long been proposed in the context of geoengineering as a way to alter Earth’s albedo^26^. These proposals have been scientifically controversial and controlled experiments encountered substantial opposition^27^. Mega-constellations will begin this process as an uncontrolled experiment^28^.

Rocket launches themselves affect the atmosphere. While cumulative CO_2_ emissions are small compared to other sources, CO_2_ is not the relevant metric. Black carbon produced by kerosene-fueled rockets such as SpaceX’s Falcon 9 and alumina particles produced by solid-fueled rockets lead to instantaneous radiative forcing. Modelling of the cumulative effect of emissions from 1000 annual launches of hydrocarbon-fuelled rockets found that, after one decade, the black carbon would result in radiative forcing comparable to that resulting from sub-sonic aviation^29^. Although 1000 launches annually is 10 times the current rate, the construction and renewal of multiple mega-constellations will require dramatic increases in launches. Current launches likely cause non-negligible radiative forcing already^30^.

Rockets fueled with liquid hydrogen do not produce black carbon but require larger tanks and therefore larger rockets, with solid-fueled boosters often being used to increase payload capacity. SpaceX’s new Starship, which the company plans to use to launch 400 Starlink satellites at a time, will be fueled by methane, the combustion of which produces soot that may, like black carbon, contribute to radiative forcing. All liquid fuels will affect mesospheric cloud formation^31^, with potential climate consequences. Rockets even threaten the ozone layer by depositing radicals directly into the stratosphere^29^, with solid-fueled rockets causing the most damage because of the hydrogen chloride and alumina they contain^29^.

## Discussion

National regulators such as the FCC are assigning orbital shells to mega-constellations on a first come, first served basis, without assessing the effects on other countries. These could include making any addition of further satellites to those shells too dangerous to contemplate. This de facto occupation of orbital shells likely violates Article I of the 1967 Outer Space Treaty, which designates the exploration and use of space as “the province of all mankind” and open to all countries “without discrimination of any kind.” There is also Article II: “Outer space … is not subject to national appropriation by claim of sovereignty, by means of use or occupation, or by any other means.” Although regulators are not claiming sovereignty over orbital shells, allowing national companies to saturate them with satellites could constitute appropriation by “other means.” Lastly, Article IX requires that space activities be conducted “with due regard to the corresponding interests of other States”.

Mega-constellation operators and their regulators could respond that they are exercising the right to explore and use space without discrimination, the use of an orbital shell is time-limited as a result of the license, and the satellites will be actively de-orbited^32^. They could also reference that countries have been using slots in geostationary orbit for decades, resulting in the de facto exclusion of others from any given slot without this being considered appropriation. However, the use of slots in geostationary orbit is mediated by the International Telecommunications Union (ITU), which does not play the same role in LEO.

Another ‘land rush’ is occurring over radio spectrum. The ITU is involved in the allocation of frequencies to communications satellites. Under its binding instruments, countries must treat frequencies as limited resources to which others have equitable access, and therefore limit their own use. But companies are not party to those instruments and do not deal directly with the ITU. They apply for and obtain licenses from their national regulator, which early in the planning process files a general description of the mega-constellation with the ITU, including the frequencies it will use^33^. A company is required to coordinate with any satellite system that might be affected by its planned mega-constellation, provided the other system was filed before its filing, but there is no requirement to coordinate with those whose filings are made after its own. The ITU recently adopted a tiered management approach, whereby listing a mega-constellation in its ‘Master Register’ depends on certain milestones being met. This deters companies from filing and effectively claiming orbital shells years before they are ready to launch, but thereby disadvantages smaller companies and exacerbates long-term equity concerns for those developing countries that are not yet active in space.

No binding international rules exist on other aspects of mega-constellations. In 2007, the Inter-Agency Space Debris Coordination Committee (IADC), currently representing 13 space agencies, indicated that direct re-entry at the end of a satellite’s operational life was preferred but nevertheless only recommended that deorbiting conclude within 25 years. This widely accepted guideline is poorly suited for mega-constellations made up of thousands of satellites with short operational lives. It also overlooks placement, with satellites at higher altitudes producing relatively high collision probabilities when de-orbiting timescales are long^34^.

The IADC also recommended collision avoidance and end-of-life deorbiting technologies. These add costs, and in 2017 the IADC reported that adherence to its guidelines was “insufficient and no apparent trend towards a better implementation is observed”^35^. More recent analyses indicate that compliance with the end-of-life guidelines is now improving by some metrics^36^. However, these improvements appear to be driven, at least in part, by SpaceX’s own practices, which may not be followed by other mega-constellation operators. Guidelines allow for ‘free riding’, whereby individual actors can save costs through non-compliance while benefitting from the compliance of others. In the context of any shared resource, free riding can lead to a ‘tragedy of the commons,’ which is exactly what needs to be avoided in LEO.

Finally, we would be remiss not to mention the threats posed by mega-constellations to astronomy, although for a detailed discussion we refer to other recent work^37–41^. Briefly, astronomers pushed for reductions in the number and brightness of Starlink satellites after an image from a telescope in Chile was ruined. SpaceX responded by adding visors to the satellites, which has reduced their naked-eye visibility while still leaving them bright to telescopes^39^. Next generation sky surveys and observations close to the horizon, especially near sunrise and sunset, are especially vulnerable—and critical for near-Earth object observations for planetary defence. Occultations are another issue: even a satellite that is unilluminated (i.e. passing through the shadow of the Earth) can interfere with rapid time domain astronomy when it passes in front of a star. Radio astronomy is also threatened^39^, since mega-constellations will require frequencies additional to those traditionally used by land stations. These could encroach on protected spectrum through out-of-band overtone emission. The large number of fast-moving transmitting stations (i.e. satellites) will cause further interference. New analysis methods could mitigate some of these effects, but data loss is inevitable, increasing the time needed for each study and limiting the overall amount of science done.

There are reasons for hope. SpaceX is showing some leadership with rapid end-of-life deorbiting, automatic collision avoidance, and visors to reduce light pollution, even if these are not yet sufficient. Spacefaring countries, moreover, recognize that debris threatens all satellites, including military satellites. Some are strengthening their national regulations, including by incorporating non-binding international guidelines into binding national laws. However, there is little recognition that Earth’s orbit is a finite resource, the space and Earth environments are connected, and the actions of one actor can affect everyone. Until that changes, we risk multiple tragedies of the commons in space.

## Methods

Figure 1 is produced using data obtained from the USSPACECOM satellite catalogue ^14^ and cross-referencing with on-orbit fragmentation records^42^. All orbits are included. Sudden rises in the CPDF are typically due to fragmentation events, while decreases are driven by orbital decay.

Figure 2, right panel and Fig. 3 are constructed by using apogee-perigee information from the satellite catalogue, which is then used to determine osculating Keplerian orbits. Those orbits are used, in turn, for assigning a given space object’s contribution to each orbital shell’s object density. All shells are averaged over the entire sky ($4\uppi$), so inclination information is not used. A satellite’s contribution to the number density within a specific shell is computed by the fraction of time per orbit the satellite spends traversing that shell. For example, if the satellite spends half of its time in a shell, then it only contributes 0.5 satellites to the total satellite count in that shell. The satellite number density in a shell is just the weighted satellite count divided by the shell volume. We use 1 km radial widths for all shells. It should be recognized that orbital inclinations for satellites will lead to local variations in the actual satellite volume density for any given point in space. In some cases, this will be much higher than the all-sky average, and in some cases, much lower.

In setting the mega-constellation number densities for Fig. 3, we assume that the constellation satellites have an eccentricity of $2\times {10}^{-4}$, which is based on the typical apogee-perigee altitudes for Starlink satellites above 500 km, as given in the satellite catalogue. The Starlink contributions are given according to FCC filed and approved altitudes (335.9, 340.8, 345.6, 550, 1110, 1130, 1275, and 1325 km) and corresponding numbers (2493, 2478, 2547, 1584, 1600, 400, 375, 450). The OneWeb constellation is assumed to consist of 6372 satellites at 1200 km (as filed). It should be recognized that this is an ongoing process, and further changes to satellite orbits and numbers may be filed. As noted in the main text, SpaceX has already filed for an additional 30,000 satellites. It is also seeking to lower the 1000 km shells to the 500–600 km region^43^.

We construct a simple collision estimate between Starlink satellites and untracked debris in the 550 km shell as follows: We take the typical relative speed between any two random objects in a shell to be $$v\approx 10$$ km/s, which assumes randomly oriented circular orbits. We let the cross-sectional area *A* = 10 m^2^, which is a rough estimate that includes the body and solar panels. Specifying that the debris is untracked means that collision avoidance is not possible, at least with full knowledge of the debris. In this case, the collision rate between a single debris particle and any satellite in the shell is *R* = *n v A*. The probability that one or more collisions will occur during some time *t* is $$P=1-\mathit{exp}\left(-\tau \right)$$, where $$\tau$$ is the total effective optical depth given by *R t* over all relevant debris particles. For the above values and satellite $$n\approx {10}^{-6}{\text{ km}}^{-3}$$, we find $${\uptau }_{i}=0.003$$ for the i-th debris particle. If all debris particles are independent, then $$P \approx \raise.5ex\hbox{$\scriptstyle 1$}\kern-.1em/ \kern-.15em\lower.25ex\hbox{$\scriptstyle 2$}$$ after 1 year for about 230 pieces of debris (i.e., $$\tau \approx 0.7$$). Given the large number of debris pieces in orbit above 550 km due to the 2007 Chinese ASAT test and the 2009 Kosmos 2251–Iridium 33 collision, significant debris should be expected to be decaying through the mega-constellation shells at any given time.

Another estimate for the collision risk with untracked debris can be made by looking at the actual debris distribution, which has an all-sky averaged density of about ${n}_{deb}(>10)\approx 7\times {10}^{-9}{\text{ km}}^{-3}$ at 550 km (see Fig. 2). This is only the catalogued and tracked debris, which is again approximately representative of objects with diameters $D\gtrsim 10\;{\text{cm}}$. The total amount of debris in LEO of that size scale is 12,400 (excluding rocket bodies and payloads). Statistical estimates for sizes $D\gtrsim 1\;{\text{cm}}$ suggest a population of $9\times {10}^{5}$ objects^44^. While the smaller size scales need not correspond directly to the catalogued debris population, we can, for the moment, assume that they do.

Under these approximations, the number density of untracked debris in the 550 km shell is 70 × the catalogued density, i.e., ${n}_{deb}\left(>1\right)\approx 5\times {10}^{-7}{\text{ km}}^{-3}$. In this situation, the collision rate between satellites and untracked debris is ${R}_{deb}={n}_{deb}\left(>1\right)A{N}_{sat}v$, where ${N}_{sat}$ = 1584, the number of satellites in the 550 km shell. Using the same values for *A* and *v*, we find ${R}_{deb}t\approx 2.5$ for *t* = 1 year, which means that there is a 92% chance of having one or more debris collisions during that time.

The reported meteoroid flux estimate using the Grün model includes an extra factor of 4 to account for gravitational focusing and no tumbling. A total satellite cross-section of A = 10 m^2^ is again assumed.
